# Supplementary material for: Strain-Level Dynamics Reveal Regulatory Roles in Atopic Eczema by Gut Bacterial Phages
Source: Microbiol Spectr. 2023 Mar 23;11(2):e04551-22. doi: 10.1128/spectrum.04551-22 (PMC10101075; doi:10.1128/spectrum.04551-22)
Supplement: Supplemental file 1 — Supplemental material. Download spectrum.04551-22-s0001.pdf, PDF file, 4.5 MB [file spectrum.04551-22-s0001.pdf]

Fig S1

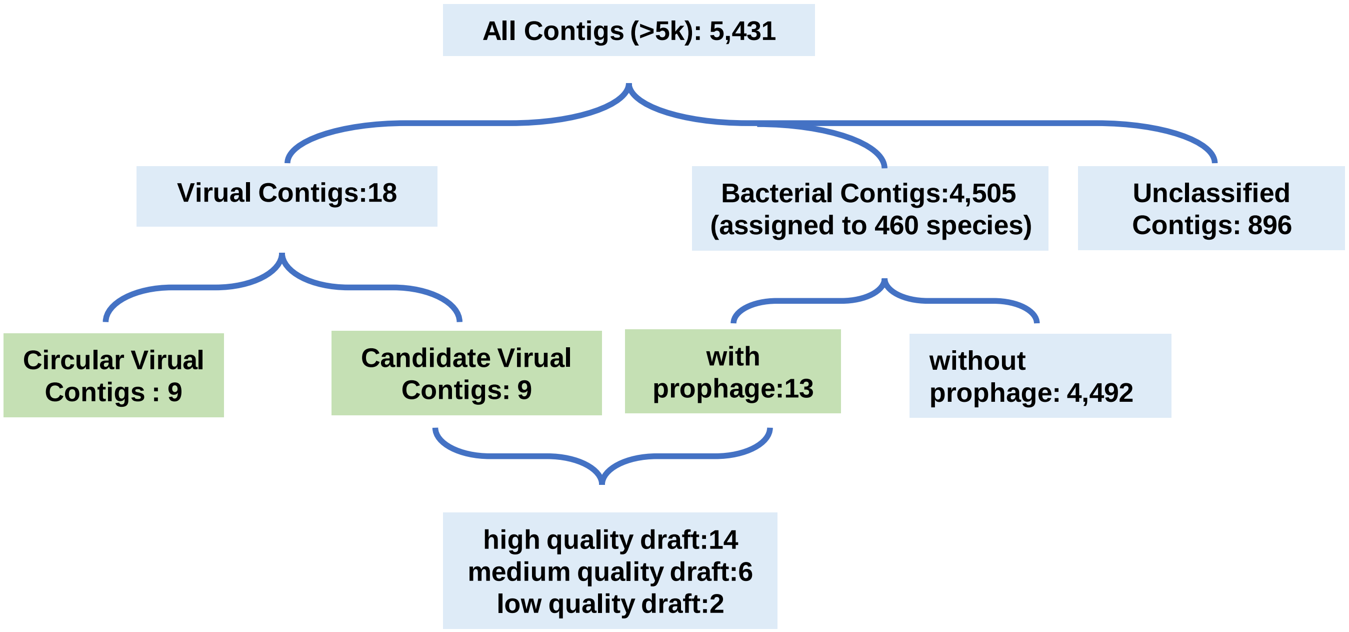

Fig S1. The assembly and annotation of the PRM for the reported case

Fig S2

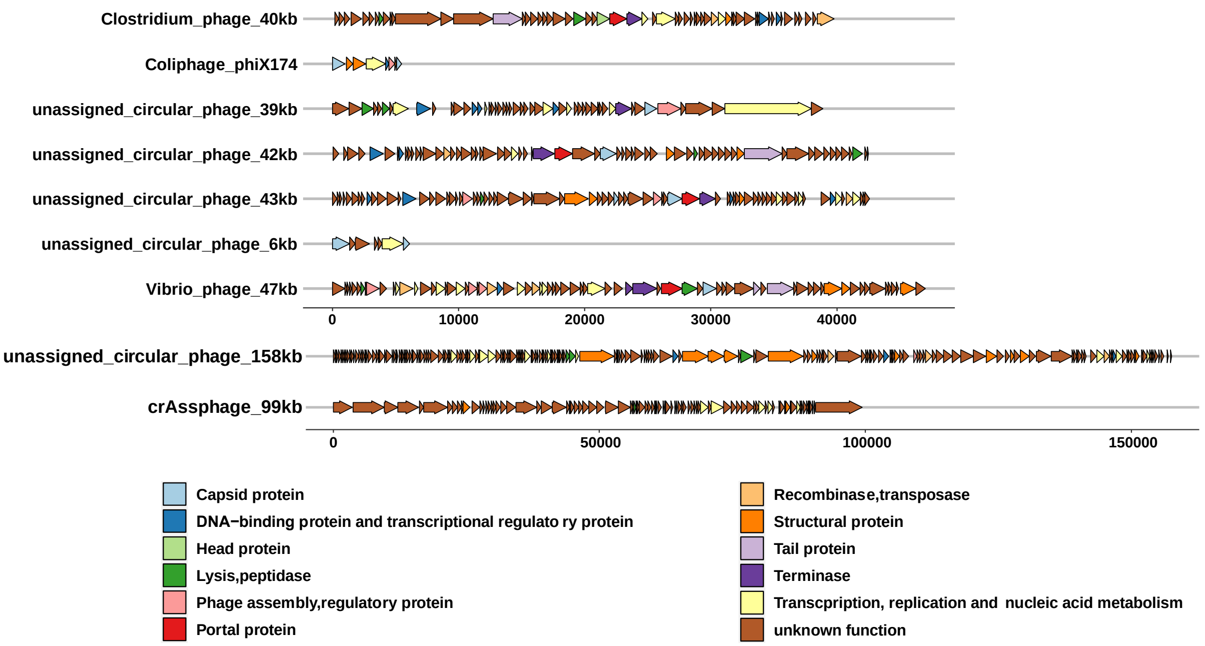

Fig S2. The structure of the completed circular phage genomes.

Fig S3

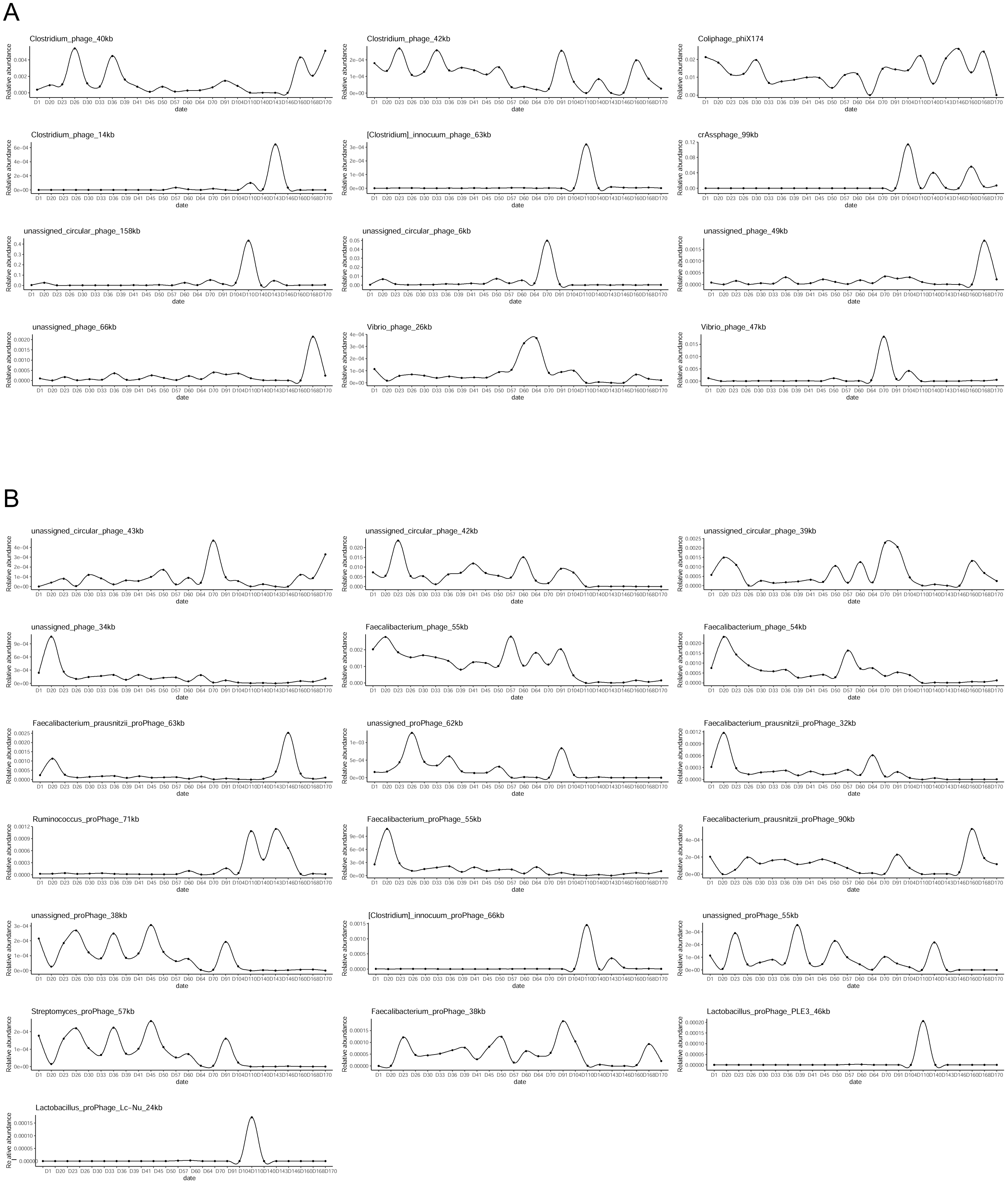

Fig S3. The dynamics relative abundance of the 31 viruses.
A. lytic phages (12 strains) . B. lysogenic phages (19 strains).

Fig S4

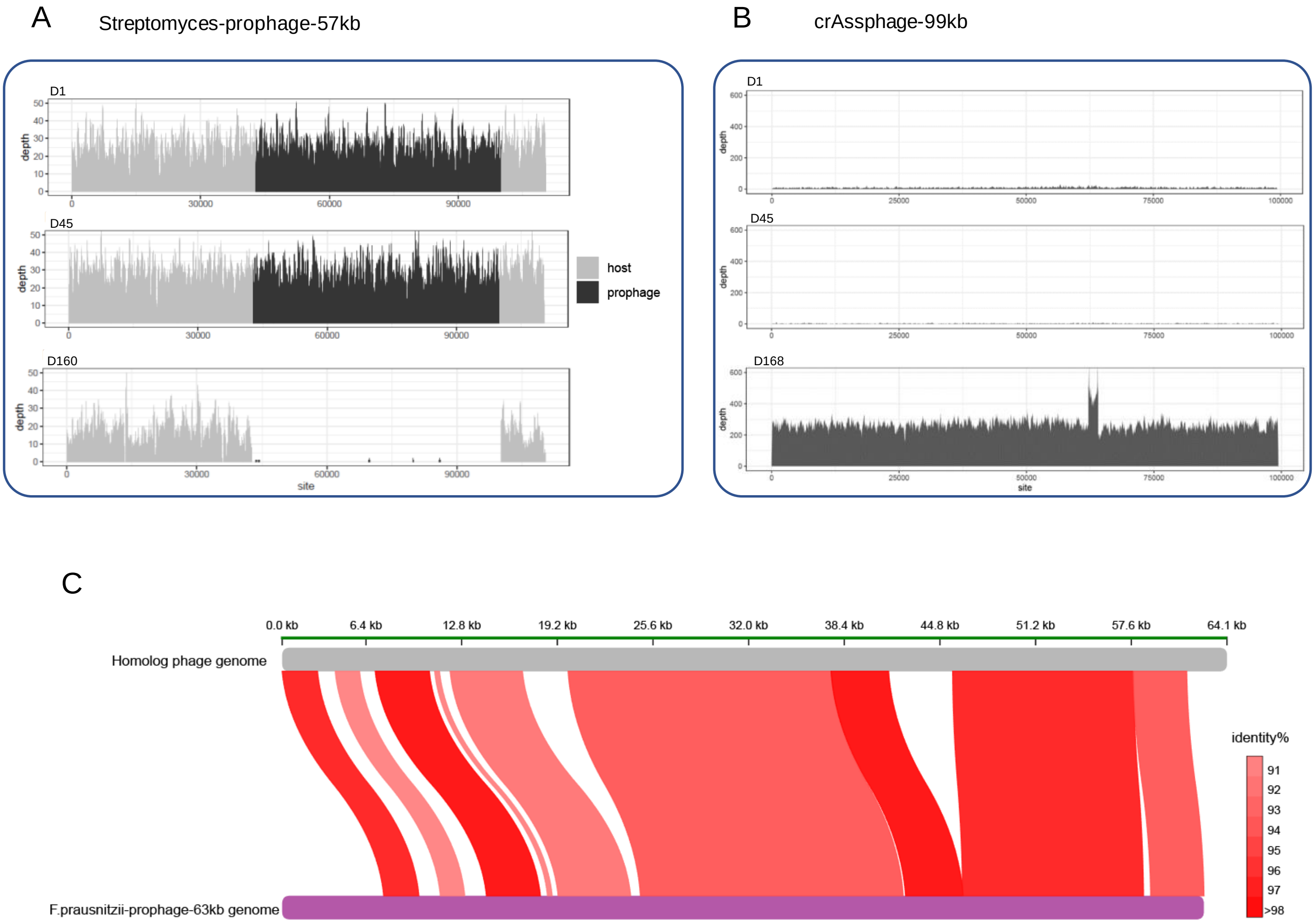

Fig S4. Illustrations of in-and-out and substitution events of viruses during the observation course. **A**. The coverage of mapping reads to the contig containing Streptomyces-proPhage-57kb in various time points exhibiting the depletion of this virus from the flora. **B**. The The coverage of mapping reads to the contig of crAssphage-99kb in various time points showing its explosive flourishment during the observation. **C.** Comparison between the genome of F.prausnitzii-prophage-63kb (the lower purple line) to its homolog phage (the upper grey line), red lines between the genomes link homologous regions of the two phages.

Fig S5

A

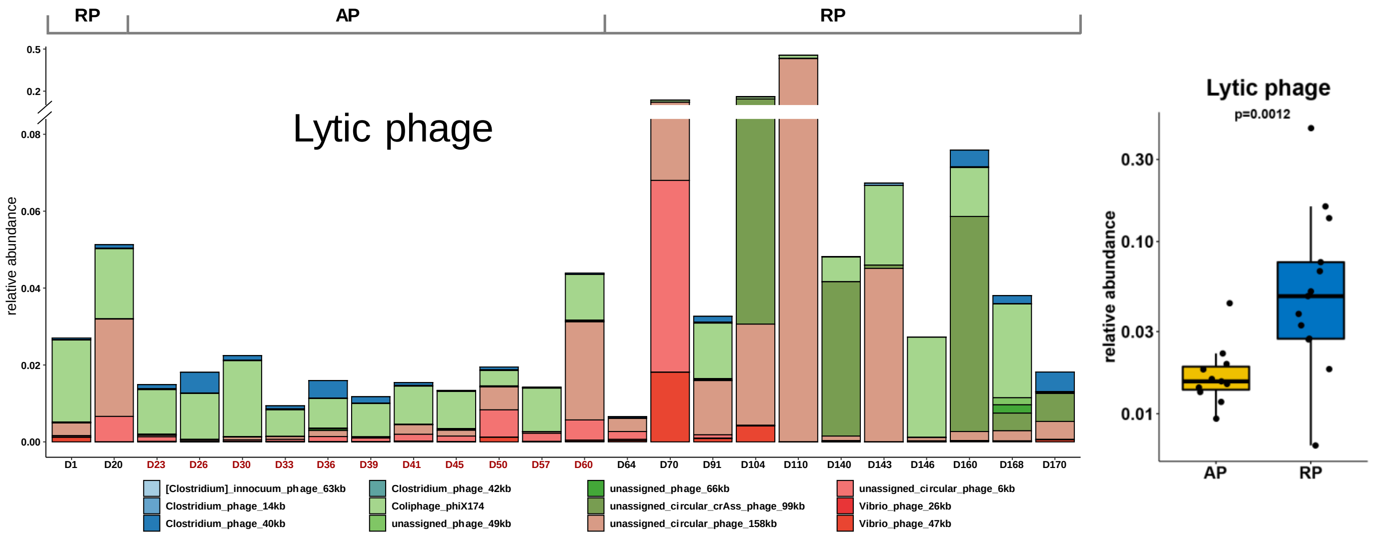

B

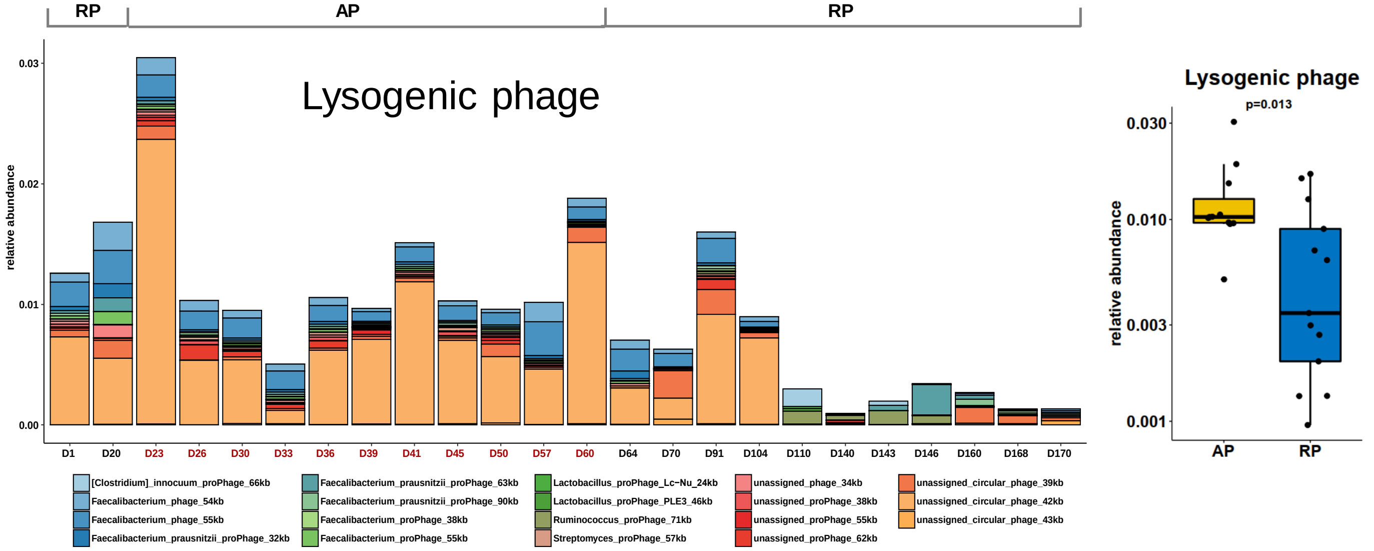

Fig S5. The relative abundance of total lytic (A) and lysogenic (B) phages during the observation course. On the left panel, each virus is marked with a different color and the horizontal axis indicates the days when samples are collected, where the samples are divided into AP and RP, highlighted in red and black, respectively. AP, active phase; RP, remission phase. The right panels indicate comparisons of the relative abundance of lytic (A) and lysogenic (B) phages between AP and RP samples. Significance was tested with Mann-Whitney U test. In box-whisker plots, the middle horizontal line refers to median value, and width of box is interquartile range (IQR) with 1.5IQR whisker length.

Fig S6

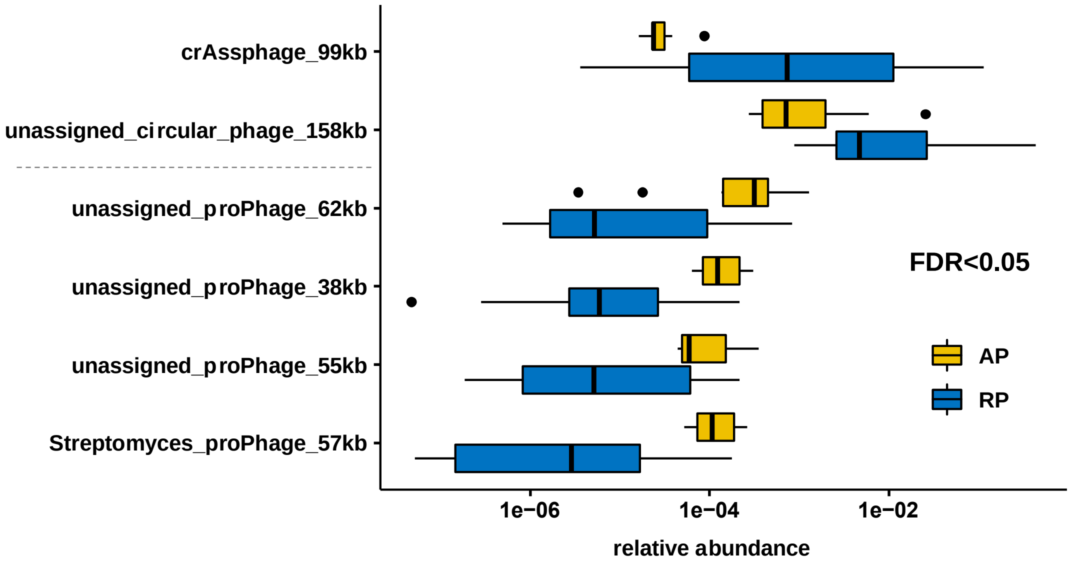

Fig S6. The differential phages (the upper two) and prophages (the lower four) between the samples of AP and RP. The significance of differences in abundance were tested by Mann-Whitney U test for each virus with p-values adjusted by Benjamin and Hochberg's approach. FDR < 0.05 was considered to be significant. In box-whisker plots, the middle horizontal line refers to median value, and width of box is interquartile range (IQR) with 1.5IQR whisker length.

Fig S7

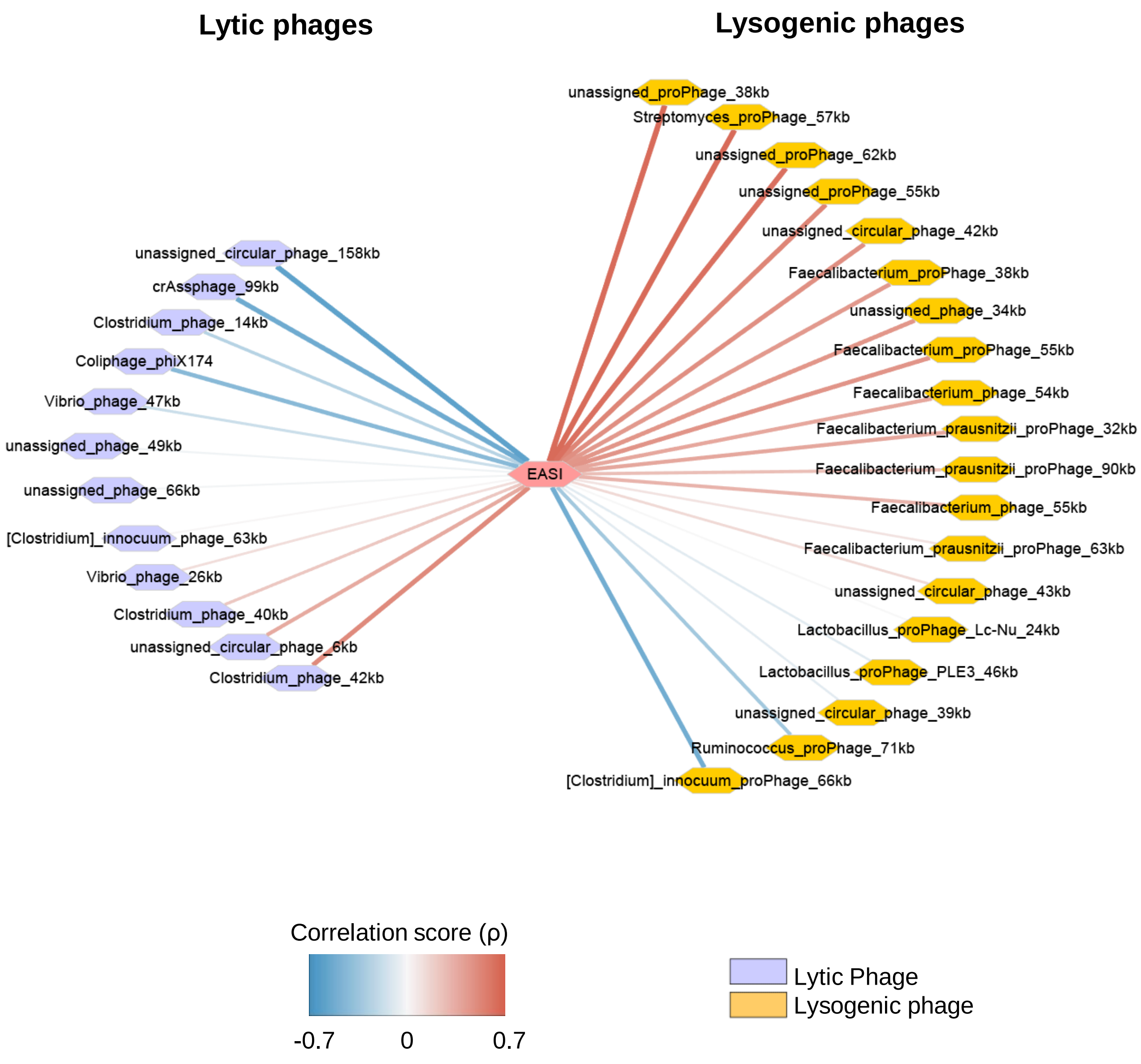

Fig S7. Correlations between phages abundance and EASI score. (Spearman's rank correlation test)

Fig S8

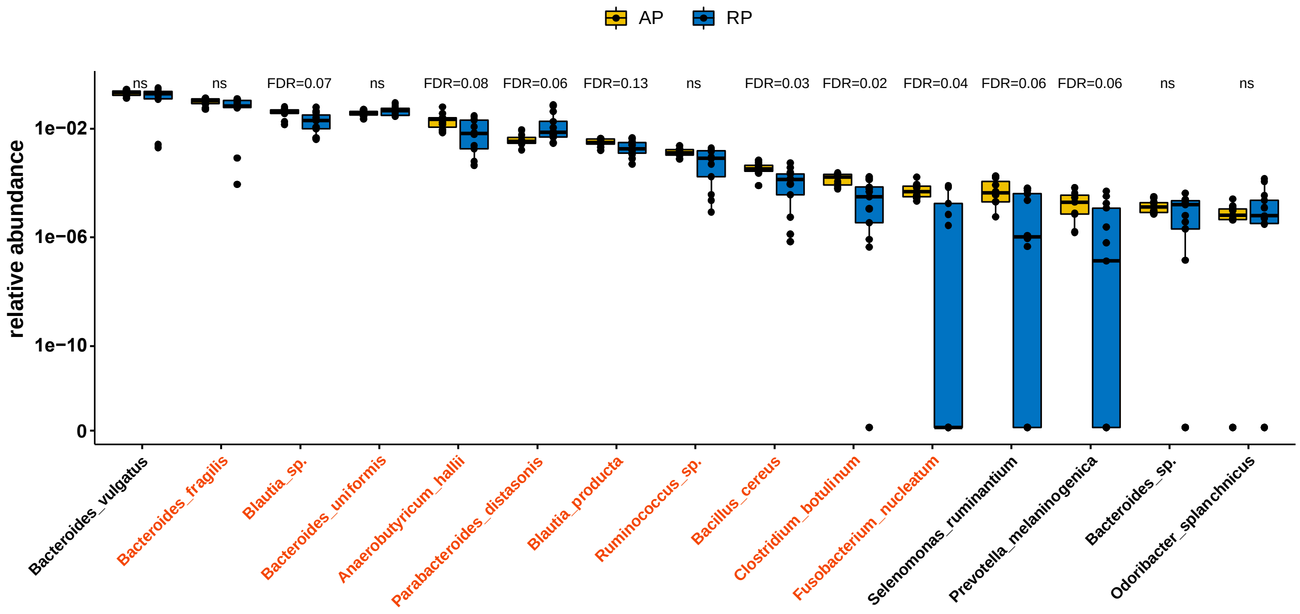

Fig.S8 The comparison of relative abundance of candidate crAssphage's host between AP and RP. The significance of differences in abundance were tested by Mann-Whitney U test for each species with p-values adjusted by Benjamin and Hochberg's approach. In box-whisker plots, the middle horizontal line refers to median value, and width of box is interquartile range (IQR) with 1.5IQR whisker length. AAA-metabolizing species were highlighted in orange.

Fig S9

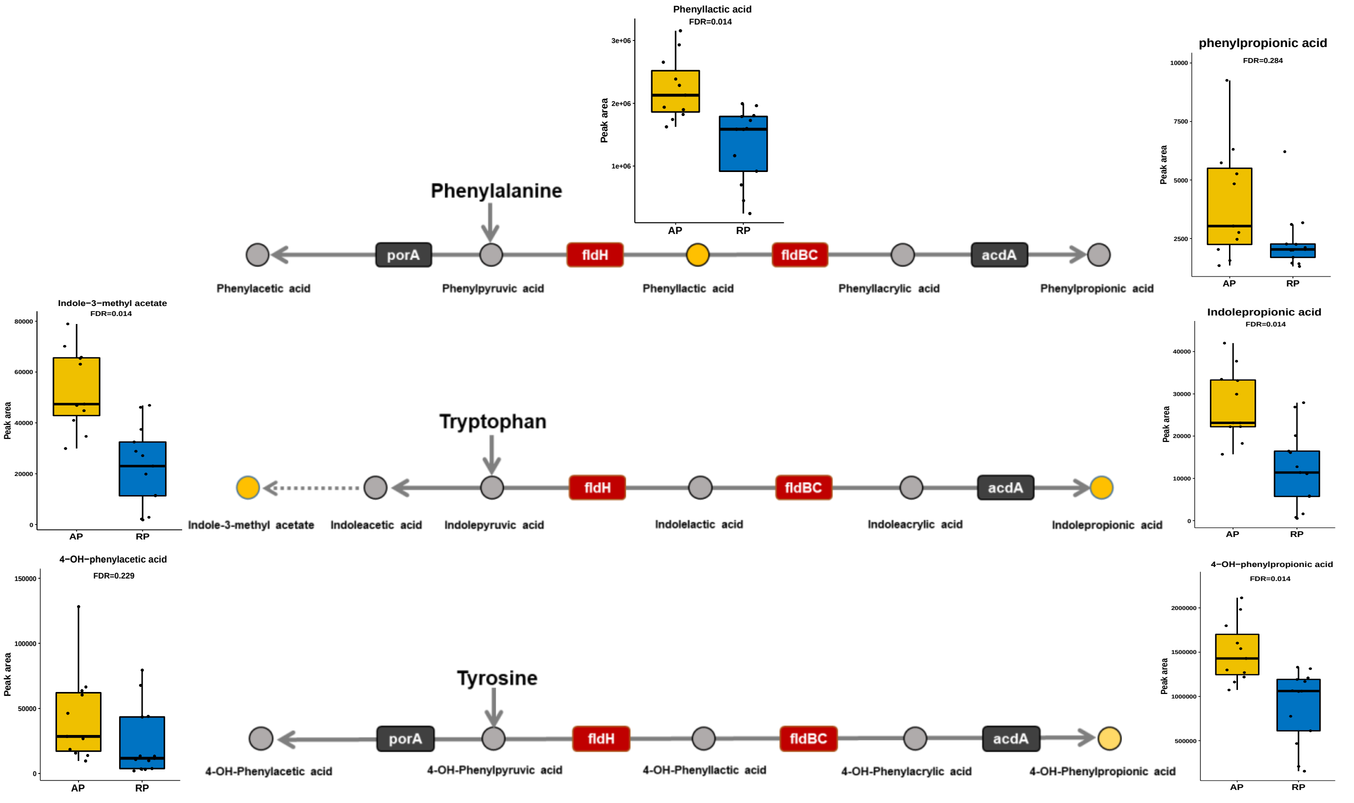

Fig S9. The schematic of AAA metabolism pathway in gut bacteria. Solid circles represent metabolites. Solid circles colored in yellow refer to metabolites increased significantly in AP samples, and those colored in grey refer to metabolites that were not significantly different between the two phases. Solid boxes with gene name refer to the key enzymes gene in each pathway. Red box refer to gene increased significantly in AP samples, and those colored in grey refer to genes that were not significantly different between the two phases. Box plots indicate the comparison of the metabolites concentrations between the two phases. The significance of differences was tested by Mann-Whitney U test with P-values adjusted by Benjamin and Hochberg’s approach.

Fig S10

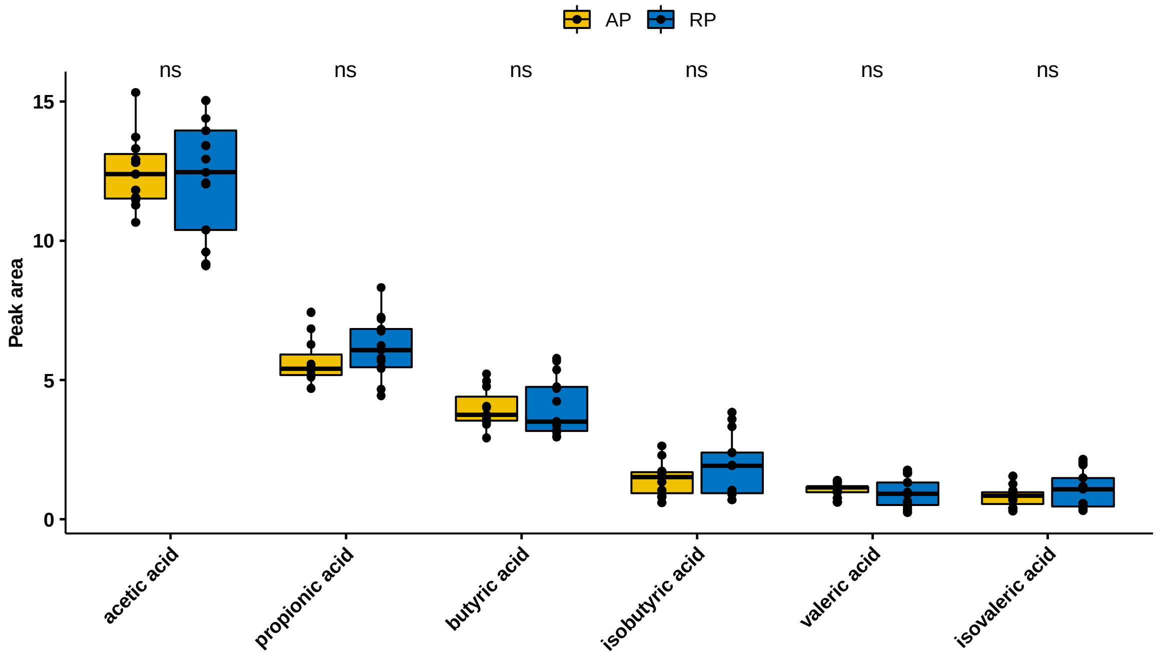

Fig S10. The comparison of SCFA concentrations between AP and RP. The significance of differences was tested by Mann-Whitney U test with p-values adjusted by Benjamin and Hochberg's approach. NS refers to nosignificant difference. In box-whisker plots, the middle horizontal line refers to median value, and width of box is interquartile range (IQR) with 1.5IQR whisker length.

Supplementary Information for

Strain-level Dynamics Reveals Regulatory Roles in Atopic Eczema by Gut Bacterial Phages

Yanan Chu[1#], Qingren Meng[2#], Jun Yu[3], Juan Zhang[4], Jing Chen[1], Yu Kang[1*]

1. Beijing Institute of Genomics, Chinese Academy of Sciences/China National Center for Bioinformation, Beijing, 100101, China.
2. School of Medicine, Southern University of Science and Technology, Shenzhen 518055, China.
3. University of Chinese Academy of Sciences, Beijing 100190, China.
4. Department of Pediatric, Peking University Third Hospital, Beijing 100191, China

# These authors contribute equally.
* Correspondence to: Prof. Yu Kang, CAS Key Laboratory of Genome Sciences and Information, Beijing Institute of Genomics Chinese Academy of Sciences & China National Center for Bioinformation, Beijing, 100101, China.
E-mail: kangy@big.ac.cn

This section includes:

Supplementary Tables S1 to S8
SI References

Supplementary Table S1. Information of samples in this study.

ID	Date	phases[#]	EASI score	Hiseq data size	nanopore data size	metabolome and SCFA
D1	20190120	RP	0	9746430764	-	√
D20	20190208	RP	0	8128542484	-	√
D23	20190211	AP	20.2	8184076198	-	√
D26	20190214	AP	19.9	9003717772	-	√
D30	20190218	AP	7.4	9183274884	-	√
D33	20190221	AP	20.3	8601365366	-	√
D36	20190224	AP	8	7775052894	-	√
D39	20190227	AP	13.4	9490718374	-	√
D41	20190301	AP	18.5	8027477988	-	√
D45	20190305	AP	11.6	8861317342	-	√
D50	20190310	AP	12.6	7433991500	4963495923	√
D57	20190317	AP	6	7793923812	-	√
D60	20190320	AP	11	7525764156	-	√
D64	20190324	RP	0	17356824708	-	√
D70	20190330	RP	0	7295166538	-	√
D91	20190420	RP	0	8188317840	-	√
D104	20190503	RP	0	9547288986	-	√
D110	20190509	RP	0	7213268594	4961309089	√
D140	20190608	RP	0	10037455672	-	√
D143	20190611	RP	0	10247377264	-	√
D146	20190614	RP	0	9380286268	-	√
D160	20190628	RP	0	8476846370	-	√
D168	20190706	RP	0	6865208698	-	√
D170	20190708	RP	0	12668732782	-	√

[#] AP, active phase; RP, remission phase

Supplementary Table S2. Recording and calculation# of EASI score.

	Body region	Redness	Thickness	Scratching	Lichenification	Severity score	Area score	Multiplier	Region score	final score
D23	Head/neck	3	0	3	0	6	3	0.2	3.6	20.2
	Trunk	1	0	0	1	2	1	0.3	0.6	
	Upper limbs	3	1	3	1	8	4	0.2	6.4	
	Lower limbs	3	1	3	1	8	4	0.3	9.6	
	Body region	Redness	Thickness	Scratching	Lichenification	Severity score	Area score	Multiplier	Region score	final score
D26	Head/neck	2	0	2	0	4	2	0.2	1.6	19.9
	Trunk	1	0	0	0	1	1	0.3	0.3	
	Upper limbs	3	2	3	1	9	4	0.2	7.2	
	Lower limbs	3	2	3	1	9	4	0.3	10.8	
	Body region	Redness	Thickness	Scratching	Lichenification	Severity score	Area score	Multiplier	Region score	final score
D30	Head/neck	3	0	2	0	5	2	0.2	2	7.4
	Trunk	0	0	0	0	0	0	0.3	0	
	Upper limbs	1	0	1	1	3	3	0.2	1.8	
	Lower limbs	1	1	1	1	4	3	0.3	3.6	
	Body region	Redness	Thickness	Scratching	Lichenification	Severity score	Area score	Multiplier	Region score	final score
D33	Head/neck	2	0	3	0	5	2	0.2	2	20.3
	Trunk	1	0	0	0	1	1	0.3	0.3	
	Upper limbs	3	2	3	1	9	4	0.2	7.2	
	Lower limbs	3	2	3	1	9	4	0.3	10.8	
	Body region	Redness	Thickness	Scratching	Lichenification	Severity score	Area score	Multiplier	Region score	final score
D36	Head/neck	2	0	2	0	4	2	0.2	1.6	8
	Trunk	1	0	0	0	1	1	0.3	0.3	

	Body region	Redness	Thickness	Scratching	Lichenification	Severity score	Area score	Multiplier	Region score	final score
	Upper limbs	2	0	1	1	4	2	0.2	1.6	
	Lower limbs	2	1	1	1	5	3	0.3	4.5	
D39	Body region	Redness	Thickness	Scratching	Lichenification	Severity score	Area score	Multiplier	Region score	final score
	Head/neck	2	0	2	1	5	2	0.2	2	13.4
	Trunk	1	1	0	0	2	1	0.3	0.6	
	Upper limbs	2	2	1	1	6	3	0.2	3.6	
	Lower limbs	2	2	1	3	8	3	0.3	7.2	
D41	Body region	Redness	Thickness	Scratching	Lichenification	Severity score	Area score	Multiplier	Region score	final score
	Head/neck	3	1	3	0	7	4	0.2	5.6	18.5
	Trunk	1	0	0	0	1	1	0.3	0.3	
	Upper limbs	3	2	3	1	9	3	0.2	5.4	
	Lower limbs	3	2	2	1	8	3	0.3	7.2	
D45	Body region	Redness	Thickness	Scratching	Lichenification	Severity score	Area score	Multiplier	Region score	final score
	Head/neck	2	1	2	0	5	2	0.2	2	11.6
	Trunk	0	0	0	0	0	0	0.3	0	
	Upper limbs	3	2	3	1	9	3	0.2	5.4	
	Lower limbs	2	2	2	1	7	2	0.3	4.2	
D50	Body region	Redness	Thickness	Scratching	Lichenification	Severity score	Area score	Multiplier	Region score	final score
	Head/neck	3	1	2	0	6	2	0.2	2.4	12.6
	Trunk	0	0	0	0	0	0	0.3	0	
	Upper limbs	3	2	3	1	9	3	0.2	5.4	
	Lower limbs	2	3	2	1	8	2	0.3	4.8	
D57	Body region	Redness	Thickness	Scratching	Lichenification	Severity score	Area score	Multiplier	Region score	final score
	Head/neck	1	1	2	0	4	2	0.2	1.6	6
	Trunk	0	0	0	0	0	0	0.3	0	
	Upper limbs	1	2	1	1	5	2	0.2	2	
	Lower limbs	2	0	1	1	4	2	0.3	2.4	

	Body region	Redness	Thickness	Scratching	Lichenification	Severity score	Area score	Multiplier	Region score	final score
D60	Head/neck	3	1	3	0	7	3	0.2	4.2	11
	Trunk	0	0	0	0	0	0	0.3	0	
	Upper limbs	2	2	3	1	8	2	0.2	3.2	
	Lower limbs	2	1	2	1	6	2	0.3	3.6	

[#] **Calculation Formula(1)**:

**Body region**	**Redness**	**Thickness**	**Scratching**	**Lichenification**	**Severity score**	**Area score**	**Multiplier**	**Region score**
Head/neck	________	+________	+________	+________	=________	X________	X 0.2	=________
Trunk	________	+________	+________	+________	=________	X________	X 0.3	=________
Upper limbs	________	+________	+________	+________	=________	X________	X 0.2	=________
Lower limbs	________	+________	+________	+________	=________	X________	X 0.3	=________
				**The final EASI score: add up the 4 region scores**				=________ (0-72)

Supplementary Table S3. Information of contigs(>1k) assembled with solely short-reads and combined reads.

Assembly	Number of contigs	N50 length	The max length	The full length
D1	33,158	14,059	693,916	160,550,972
D20	21,619	12,971	526,128	94,157,209
D23	27,718	14,892	694,005	128,549,275
D26	28,530	10,421	726,825	118,741,777
D30	35,977	6,385	693,916	132,956,807
D33	32,150	14,527	728,087	142,295,850
D36	22,325	16,913	694,248	103,929,533
D39	25,272	15,110	992,793	107,310,383
D41	25,363	7,591	726,825	99,706,791
D45	30,529	11,598	728,053	141,835,651
D57	22,968	9,495	693,916	93,272,404
D60	23,072	12,871	1,415,749	98,164,056
D64	30,890	18,422	693,916	137,684,500
D70	21,885	8,854	1,415,474	86,097,854
D91	28,217	17,000	1,415,623	136,859,952
D104	24,599	16,828	793,917	117,599,303
D140	27,696	6,869	765,220	106,947,069
D143	8,636	45,734	727,068	52,598,549
D146	15,748	17,871	992,793	81,770,739
D160	30,120	10,167	694,411	131,726,984
D168	29,368	6,600	879,134	115,057,699
D170	30,983	23,670	876,735	154,451,081
D50- GridION	30,935	10,444	678,149	142,624,078
D110-GridION	4,395	34,830	726,461	28,149,221
D50(Hiseq+GridION)	25,026	24,467	1,450,244	152,759,008
D110(Hiseq+GridION)	2,349	92,321	2,796,291	32,732,314
PRM (contigs>5k)	5,431	71,829	2,796,291	140,768,589

Supplementary Table S4. The list of phage strains identified.

VIBRANT							checkV		
phage/ prophage	name	length	type	quality	GC	genes	checkV quality	completeness	contamination
phage	[Clostridium]_innocuum_phage_63kb	62677	lytic	high	0.41	66	high	90.21	0
	Clostridium_phage_14kb	13989	lytic	low	0.29	21	low	25.41	0
	Clostridium_phage_40kb	39812	lytic	complete	0.42	60	complete	100	0
	Clostridium_phage_42kb	42434	lytic	medium	0.43	58	high	100	9.12
	Coliphage_phiX174	5487	lytic	complete	0.45	8	complete	100	0
	unassigned_phage_49kb	49067	lytic	high	0.32	73	medium	62.6	0
	unassigned_phage_66kb	65625	lytic	high	0.32	103	medium	83.41	0
	crAssphage_99kb	99416	lytic	complete	0.29	94	high	93.52	0
	unassigned_circular_phage_158kb	157775	lytic	complete	0.42	195	high	100	0
	unassigned_circular_phage_6kb	6108	lytic	complete	0.40	7	complete	100	0
	Vibrio_phage_26kb	26428	lytic	low	0.59	42	low	48.36	0
	Vibrio_phage_47kb	47009	lytic	complete	0.63	73	complete	100	0
	Faecalibacterium_phage_54kb	54586	lysogenic	medium	0.58	79	high	92.08	0
	Faecalibacterium_phage_55kb	54966	lysogenic	medium	0.54	83	high	100	0
	unassigned_phage_34kb	33770	lysogenic	high	0.56	54	medium	76.57	21.95
	unassigned_circular_phage_39kb	38878	lysogenic	complete	0.50	51	complete	100	0
	unassigned_circular_phage_42kb	42481	lysogenic	complete	0.47	65	complete	100	0
	unassigned_circular_phage_43kb	42594	lysogenic	complete	0.51	78	complete	100	0
prophage	[Clostridium]_innocuum_proPhage_66kb	65997	lysogenic	high	0.42	71	high	100	26.89
	Faecalibacterium_prausnitzii_proPhage_32kb	32153	lysogenic	high	0.60	45	high	92.16	0
	Faecalibacterium_prausnitzii_proPhage_63kb	62557	lysogenic	high	0.55	104	medium	84.42	16.71
	Faecalibacterium_prausnitzii_proPhage_90kb	89943	lysogenic	high	0.51	97	high	92.64	49.52
	Faecalibacterium_proPhage_38kb	38235	lysogenic	medium	0.45	59	high	98.99	0
	Faecalibacterium_proPhage_55kb	54816	lysogenic	medium	0.52	78	medium	69.28	23.81
	Lactobacillus_proPhage_Lc-Nu_24kb	24142	lysogenic	medium	0.43	30	medium	61.12	0
	Lactobacillus_proPhage_PLE3_46kb	45729	lysogenic	high	0.45	71	high	95.99	13.18
	Ruminococcus_proPhage_71kb	70841	lysogenic	high	0.41	98	complete	100	23.98
	Streptomyces_proPhage_57kb	57169	lysogenic	high	0.35	81	high	98.88	0
	unassigned_proPhage_55kb	54952	lysogenic	high	0.32	94	high	98.99	10.07
	unassigned_proPhage_38kb	37899	lysogenic	high	0.37	54	high	100	0
	unassigned_proPhage_62kb	62402	lysogenic	high	0.45	72	high	100	10.31

Supplementary Table S5. The comparison between PRM and MAG reference

	**PRF(>5k)**	**MAG(>5K)**
The contigs' number	5,431	9,753
N50 length	71,829	74,150
N10 length	677,509	302,150
No. of contigs >500k	27	14
No. of contigs >1M	5	1
The max length	2,796,291	1,415,749
The full length	140,768,589	294,898,180
**VIBRANT predicts virus**		
No. of circular/complete virus	9	3
Virus total Length	479,560	109,496
High-quality virus	17	26
High-quality virus total length	1,034,433	1,346,934
Medium-low quality virus	217	198
No.of total virus fragments	234	224
Mapping rate	99.10±0.52%	85.55±0.04%

Supplementary Table S6. Correlations between phages abundance and EASI score

EASI	phage	type	ρ[#]	P-value	correlations
EASI	unassigned_circular_phage_158kb	lytic	-0.59521	0.002153	negative
EASI	crAssphage_99kb	lytic	-0.52887	0.007881	negative
EASI	unassigned_proPhage_55kb	lysogenic	0.530764	0.007621	positive
EASI	unassigned_proPhage_62kb	lysogenic	0.623648	0.001129	positive
EASI	unassigned_proPhage_38kb	lysogenic	0.64829	0.000613	positive
EASI	Streptomyces_proPhage_57kb	lysogenic	0.651134	0.000569	positive

[#]Spearman's rank correlation test, $\rho>0.5$ or <-0.5 and $P<0.01$

Supplementary Table S7. Potential hosts of crAss-like phages.

potential hosts of crAss phages*	median in RP	median in AP	P-value[#]	FDR	enriched in	potential hosts reported in	AAA-metabolizing capacity reported in
Clostridium botulinum	3.14E-05	1.66E-04	0.0015	**0.0208**	AP	Gulyaeva et al.,(2)	Liu et al.,(3)
Bacillus cereus	1.38E-04	3.35E-04	0.0039	**0.0329**	AP	Gulyaeva et al.,(2)	Prasad et al.,(4)
Fusobacterium nucleatum	0.00E+00	4.81E-05	0.0058	**0.0424**	AP	Gulyaeva et al.,(2)	Liu et al.,(3)
Prevotella melaninogenica	1.35E-07	1.99E-05	0.0142	0.0622	AP	Gulyaeva et al.,(2); Yutin et al.,(5)	-
Selenomonas ruminantium	1.06E-06	4.45E-05	0.0173	0.0687	AP	Yutin et al.,(5)	-
Blautia sp.	1.99E-02	4.01E-02	0.0184	0.0700	AP	Tomofuji et al.,(6)	Rey et al.,(7)
Anaerobutyricum hallii	6.77E-03	2.23E-02	0.0257	0.0842	AP	Yutin et al.,(5); Tomofuji et al.,(6)	Zheng et al., (8)
Blautia producta	1.80E-03	3.16E-03	0.0474	0.1290	AP	Tomofuji et al.,(6)	Rey et al.,(7)
Bacteroides fragilis	6.95E-02	1.07E-01	0.1191	0.2749	-	Gulyaeva et al.,(2); Yutin et al.,(5)	Liu et al.,(3); Zheng et al., (8)
Ruminococcus sp.	8.20E-04	1.31E-03	0.1339	0.2959	-	Yutin et al.,(5) Tomofuji et al.,(6)	Zheng et al., (8)
Bacteroides sp.	1.61E-05	1.32E-05	0.4868	0.6996	-	Gulyaeva et al.,(2); Yutin et al.,(5)	-
Bacteroides vulgatus	1.94E-01	2.17E-01	0.4940	0.6996	-	Yutin et al.,(5); Tomofuji et al.,(6); Luis et al.,(9)	
Parabacteroides distasonis	7.42E-03	3.46E-03	0.0154	0.0629	RP	Gulyaeva et al.,(2); Yutin et al.,(5); Luis et al.,(9)	Liu et al.,(3)
Bacteroides uniformis	4.66E-02	3.94E-02	0.2284	0.4540	-	Yutin et al.,(5); Luis et al.,(9)	Zheng et al., (8)
Odoribacter splanchnicus	6.24E-06	6.53E-06	0.6013	0.7361		Gulyaeva et al.,(2); Yutin et al.,(5)	-

* Species with bold name are AAA-metabolizing bacteria reported in previous studies.
[#] Mann-Whitney U test for each species with P-values adjusted by Benjamin and Hochberg's approach (FDR)

Supplementary Table S8. The correlations between differential metabolites and the severity of eczema (EASI score). (Spearman's rank correlation test)

severity of eczema	metabolites	ρ	correlations
EASI score	**Indole-3-methyl acetate**	0.69094	positive
EASI score	**Phenyllactic acid**	0.60658	positive
EASI score	**D-Phenyllactic acid**	0.60658	positive
EASI score	**4-OH-phenylpropionic acid**	0.59805	positive
EASI score	**Indolepropionic acid**	0.64165	positive
EASI score	Lenticin	0.65018	positive
EASI score	N1-Methyl-2-pyridone-5-carboxamide	0.54403	positive
EASI score	Saccharopine	-0.4691	negative
EASI score	Hypoxanthine	-0.5990	negative

SI References

1. J. M. Hanifin *et al.*, The eczema area and severity index (EASI): assessment of reliability in atopic dermatitis. EASI Evaluator Group. *Exp Dermatol* **10**, 11-18 (2001).
2. A. Gulyaeva *et al.*, Discovery, diversity, and functional associations of crAss-like phages in human gut metagenomes from four Dutch cohorts. *Cell Rep* **38**, 110204 (2022).
3. Y. Liu, Y. Hou, G. Wang, X. Zheng, H. Hao, Gut Microbial Metabolites of Aromatic Amino Acids as Signals in Host-Microbe Interplay. *Trends Endocrinol Metab* **31**, 818-834 (2020).
4. C. Prasad, V. R. Srinivasan, Tryptophan catabolism during sporulation in Bacillus cereus. *Biochem J* **119**, 343-349 (1970).
5. N. Yutin *et al.*, Analysis of metagenome-assembled viral genomes from the human gut reveals diverse putative CrAss-like phages with unique genomic features. *Nat Commun* **12**, 1044 (2021).
6. Y. Tomofuji *et al.*, Whole gut virome analysis of 476 Japanese revealed a link between phage and autoimmune disease. *Ann Rheum Dis* **81**, 278-288 (2022).
7. F. E. Rey *et al.*, Dissecting the in vivo metabolic potential of two human gut acetogens. *J Biol Chem* **285**, 22082-22090 (2010).
8. Y. Zheng *et al.*, The Role of Bacterial-Derived Aromatic Amino Acids Metabolites Relevant in Autism Spectrum Disorders: A Comprehensive Review. *Front Neurosci* **15**, 738220 (2021).
9. L. F. Camarillo-Guerrero, A. Almeida, G. Rangel-Pineros, R. D. Finn, T. D. Lawley, Massive expansion of human gut bacteriophage diversity. *Cell* **184**, 1098-1109 e1099 (2021).
